# B3GALT5 regulates renal A/B antigen levels: a determinant of prognosis and rejection in ABO-incompatible kidney transplantation

**DOI:** 10.7189/jogh.16.04254

**Published:** 2026-09-25

**Authors:** Fan Zhang, Saifu Yin, Yang Xiong, Tao Lin, Xianding Wang

**Affiliations:** 1 Department of Urology, Institute of Urology, West China Hospital, Sichuan University, Chengdu, Sichuan, China; 2 Organ Transplantation Centre, West China Hospital, Sichuan University, Chengdu, Sichuan, China

**Keywords:** kidney transplantation, ABO blood-group system, blood group incompatibility, prognosis, graft rejection, B3GALT5

## Abstract

**Background:**

ABO-incompatible (ABOi) kidney transplantation alleviates the shortage of donor kidneys. However, interventions targeting blood group antibodies alone cannot fully prevent antibody-mediated rejection. We investigated how blood group antigens affect prognosis and rejection in ABOi kidney transplantation and explored their regulatory mechanisms.

**Methods:**

We prospectively enrolled 28 consecutive ABOi kidney transplant recipient-donor pairs. We performed immunohistochemical staining for A/B antigens on intraoperative donor kidney biopsies. Graft A/B antigen expression levels were assessed in relation to post-transplant anti-A/B antibody titres, graft function, and rejection. We used transcriptome sequencing, immunohistochemistry, immunofluorescence, Western blotting, and polymerase chain reaction to preliminarily explore the regulatory mechanisms of blood group antigens.

**Results:**

We identified three expression levels of antigen A (strong, medium, and weak) and two of antigen B (strong and weak) in the grafts. Recipients of grafts with strong antigen expression showed a numerically higher proportion of immunoglobulin M (IgM) elevation. Three recipients developed blood group antibody-mediated rejection, and their graft blood group antigen levels were higher than those in other individuals (difference = 10,301.8; 95% confidence interval (CI) = 4,389.5, 14,413.2, uncorrected *P* = 0.0088). Recipients of grafts with weak antigen expression exhibited an improvement in estimated glomerular filtration rate within one month post-transplant (regression coefficient = 15.13; 95% CI = 0.172, 30.077, uncorrected *P* = 0.047). Additionally, B3GALT5, B3GNT3, and FUT9 were significantly correlated with renal blood group antigen levels, and B3GALT5 overexpression reduced blood group A antigen levels in human kidney-2 and human umbilical vein endothelial cells.

**Conclusions:**

Graft A/B antigen expression levels may be associated with anti-A/B IgM/immunoglobulin G titres, renal function, and rejection, but these findings are exploratory and require validation in larger cohorts. Furthermore, B3GALT5 shows a significant correlation with renal blood group antigen levels, and modulation of B3GALT5 can alter blood group antigen expression.

Living donor kidney transplantation has significantly expanded the donor pool for patients with end-stage renal disease. However, in approximately one-quarter to one-third of cases, the potential living donor is ABO-incompatible (ABOi) with the intended recipient [1–3]. For candidates without an ABO-compatible donor, ABOi kidney transplantation serves as an alternative [4]. A high incidence of antibody-mediated rejection (AMR) initially hindered the development of this approach [5]; nevertheless, preconditioning protocols that remove anti-donor A/B antibodies have enabled successful ABOi transplantation by overcoming the ABO blood group barrier [6–8]. Comparable long-term renal function and survival rates have been reported between ABO-compatible and ABOi kidney transplantation [9].

A low pretransplant anti-A/B titre (≤1:16 at most centres) is required to prevent AMR [10–12]. Within the first two weeks after ABOi kidney transplantation, a state of accommodation typically develops, characterised by the absence of ABO antigen–antibody interaction and preserved graft function, even with A/B antigens on the graft and possible increases in corresponding antibodies in the recipient’s blood [13]. However, for reasons that remain unclear, some grafts still fail due to AMR even when pretransplant anti-A/B titres are within the safe range [14]. Current preconditioning protocols primarily focus on pretransplant extracorporeal antibody removal targeting the recipient; whether donor graft antigens influence posttransplant graft function and clinical outcomes remains unknown.

ABO blood group antigens are glycans expressed on the vascular endothelium, collecting ducts, and distal convoluted tubules of the human kidney [15]. Previous studies have shown highly individual- and tissue-specific expression patterns of A and B antigens in human organs [15–18]. Nevertheless, no research has examined the association between A/B antigen expression levels and ABOi transplantation prognosis. In this study, we tested the hypothesis that A/B antigen expression levels in donor kidneys (free of red blood cells) vary widely and investigated their impact on posttransplant anti-A/B titre fluctuations, renal function, graft dysfunction, and graft rejection. Furthermore, we preliminarily explored the regulatory mechanisms of renal blood group antigens.

## METHODS

### Study population

We enrolled patients who underwent living donor ABOi kidney transplantation at our institution between January 2020 and December 2021 and provided informed consent. We retrospectively collected and analysed clinical data for all donor–recipient pairs. The Institutional Review Board of West China Hospital and the Health Commission of Sichuan Province approved each transplant procedure. Donors with blood group A2 or A2B, as well as recipients with pre-transplant donor-specific anti-human leukocyte antigen (HLA) antibodies (DSA), were excluded.

Pretransplant immunosuppressive therapy was started 2–4 weeks before ABOi transplantation and consisted of tacrolimus 3 mg/d, prednisone 5 mg/d, and mycophenolate mofetil 1,500 mg/d or enteric-coated mycophenolate sodium 1080 mg/d. Intravenous rituximab (200 mg) was administered to the recipient two to four weeks prior to ABO-incompatible living donor kidney transplantation. Plasma exchange was then performed according to blood group antibody titres. All recipients were required to have an immunoglobulin M (IgM) A/B antigen titre of ≤1:16 on the day of transplantation [19].

### Sample collection, paraffin sectioning, immunohistochemistry and grouping

Immediately after laparoscopic living donor nephrectomy, we perfused the kidney allograft and preserved it in histidine-tryptophan-ketoglutarate solution. During cold storage, we performed a renal biopsy using a 16-gauge Max-Core disposable core biopsy instrument (C. R. Bard, Inc., Murray Hill, New Jersey, USA) (sample notch length 19 mm, penetration depth 22 mm). The biopsy wound was then repaired with a 6-0 silk suture. We fixed fresh tissue in paraformaldehyde for over 24 hours, then dewaxed, paraffin-embedded, and sectioned it. We then performed immunohistochemistry (IHC) using anti-A antibody (1:100 dilution, ab2521) (Abcam, Cambridge, UK) and anti-B antibody (1:100 dilution, ab2524) (Abcam, Cambridge, UK) as primary antibodies, with biotinylated anti-mouse IgM as the secondary antibody. We used primary antibodies against B3GALT5 (1:100, PK23457), B3GNT3 (1:100, PA5-21988), and FUT9 (1:100, ab176794).

The same pathologist semiquantitatively evaluated the distribution of positive cells (percentages within the vascular endothelium, tubular epithelium, and glomerular capillaries) and staining intensity, blinded to the clinical outcomes (Material S1 in the **Online Supplementary Document**). Additionally, we verified this assessment of ABO antigen expression using ImageJ, version 1.54p (National Institutes of Health, Bethesda, Maryland, USA) and the Immunohistochemistry Image Analysis Toolbox, version 1.0 (The University of Nottingham, Nottingham, UK).

### Definition of clinical parameters

We measured anti-donor immunoglobulin G (IgG) and IgM titres using a gel card technique. We defined the initial anti-A/B antibody titre as the recipient’s antibody level before any immunomodulatory treatment, and the pretransplant anti-A/B antibody titre as the level on the day before kidney transplantation. We measured posttransplant anti-A/B titres on days one, three, seven, and 14, as well as at months one, three, and six after surgery. We defined titre elevation as a log_2_ increase of ≥1 in the posttransplant serum blood group antibody titre compared with the pretransplant titre. Similarly, we defined titre reduction as a log_2_ decrease of ≥1 compared with the pretransplant titre.

We calculated estimated glomerular filtration rate (eGFR) using the Chronic Kidney Disease Epidemiology Collaboration equation formula [20]. We measured serum creatinine (Scr) and eGFR before any immunomodulatory treatment, on posttransplant days one, three, seven, and 14, and at posttransplant months one, two, three, four, five, and six. We defined pretransplant Scr and pretransplant eGFR as the values measured before immunomodulatory treatment. We defined graft dysfunction as clinical symptoms, including posttransplant oliguria (<1200 mL/d) and delayed reduction in Scr. Graft rejection was defined as a clinical diagnosis of rejection based on clinical symptoms (*e.g.* oliguria or oedema) or an otherwise unexplained >50% increase in Scr within three days after transplantation, in the absence of other identifiable causes. We performed biopsies in all patients with a clinical diagnosis of graft rejection.

### Transcriptome sequencing

We subjected zero-hour biopsy samples from 19 of the 28 enrolled allografts, along with four additional adjacent normal renal tissue samples from tumour patients (used to explore intra-renal antigen regulatory mechanisms), to transcriptome sequencing. We extracted total RNA and prepared libraries according to the manufacturer’s instructions. Sequencing was performed on an Illumina platform. We then grouped samples by antigen expression level in the graft. We identified differentially expressed genes between groups with high *vs.* low antigen expression intensity using ‘DESeq2’ package in *R*, version 1.42.0 (R Core Team, Vienna, Austria). Genes with a fold change ≥2 (*i.e.* log_2_ fold change (FC) ≥ 1) and adjusted *P* ≤ 0.1) were considered significantly differentially expressed [21] (Material S2, Tables S1–3 in the **Online Supplementary Document**).

### Cell line experiments

We obtained human renal proximal tubular epithelial cell line (HK-2) and human umbilical vein endothelial cells (HUVECs) from the American Type Culture Collection. Cells were cultured in high-glucose Dulbecco’s Modified Eagle Medium supplemented with 10% foetal bovine serum and 1% penicillin-streptomycin at 37°C in a 5% carbon dioxide (CO_2_) incubator. For subculture, cells reaching approximately 100% confluence were detached using trypsin-EDTA, neutralised with complete medium, and reseeded at a ratio of 1:2–1:4. Importantly, HUVECs express blood group antigens when cultured with appropriate substrates (galactose).

Lentiviral vectors for B3GALT5 overexpression and the corresponding control were constructed and amplified by Genochem. To determine the optimal puromycin concentration for selection, we seeded HK-2 cells in the logarithmic growth phase into 12-well plates and treated them with increasing concentrations of puromycin (0–5 μg/mL) for 48 hours. We used the lowest concentration that resulted in complete cell death for subsequent selection.

We transduced HK-2 and HUVEC cells with B3GALT5-overexpressing or control lentivirus. Based on the manufacturer’s protocol, we used a multiplicity of infection of five. We included the following groups: control lentivirus, B3GALT5-overexpressing lentivirus alone, B3GALT5 lentivirus plus HitransG A, B3GALT5 lentivirus plus HitransG P, B3GALT5 lentivirus plus both HitransG A and HitransG P, and a blank control (no virus). Further, 16 hours after transduction, we replaced the medium with fresh normal medium. After 72 hours, we added puromycin at the previously determined concentration for 48 hours of selection. Once 100% of cells expressed the fluorescent marker, the puromycin concentration was halved to further expand the stable cell line. We validated the established stable cells by polymerase chain reaction (PCR) and Western blotting for B3GALT5 expression, and by immunofluorescence staining for blood group antigen expression.

We determined the ABO blood group of HK-2 and HUVEC cells using the ABO gene, whose polymorphisms are mainly located in exons six and seven. We amplified exons six and seven by PCR and subjected them to Sanger sequencing (ABI 3130 sequencer). We used the obtained sequences to assign the ABO genotype.

### Quantitative real-time PCR for B3GALT5 expression

We extracted total RNA from stable and control HK-2 cells using an RNA isolation kit. After removing genomic DNA and proteins, we purified the RNA on an adsorption column, washed it, and eluted it with RNase-free water. We performed quantitative real-time PCR to quantify B3GALT5 mRNA levels (Tables S4–8 in the **Online Supplementary Document**).

### Immunofluorescence staining for A/B blood group antigen expression

To detect A/B blood group antigen expression on the cell surface, we subjected stable B3GALT5-overexpressing HK-2 cells and control HK-2 cells, and stable B3GALT5-overexpressing HUVEC cells and control HUVEC cells to immunofluorescence staining. Cells in the logarithmic growth phase were detached, resuspended as a single-cell suspension, and seeded onto coverslips placed in culture dishes. After incubation at 37°C in 5% CO_2_ for 48–72 hours, the culture medium was removed, the cells were washed two to three times with phosphate-buffered saline (PBS), and fixed with 4% paraformaldehyde for 30 minutes. The fixed cells were then washed three times with PBS (five minutes each), blocked with 10% normal serum for 30 minutes at room temperature, and incubated with primary antibody against ABO blood group antigen (1:100, ab24223). After PBS washing, we incubated the cells with the secondary antibody for 30 minutes at 37°C, then with 4, 6-diamidino-2-phenylindole for 10 minutes at room temperature. Finally, we mounted the coverslips using an anti-fade mounting medium.

### Data analysis

We examined the association between blood group antigen expression levels and Scr and eGFR values during the first month post-transplant using generalised estimating equations (GEE) and a logistic regression model, which allowed assessment of correlations among repeated measurements within the same patient. In this model, we set Scr or eGFR as the dependent variable, and donor graft antigen expression level was an independent fixed variable.

Data are presented as mean (x̄) and standard deviation (SD) or median and interquartile range (IQR). We compared normally distributed data between groups using Student’s *t*-test, and non-normally distributed data using the Wilcoxon rank-sum test. Categorical variables were compared using the χ^2^ test or Fisher’s exact test. We visualised results using the ‘ggplot2’ package in *R*. To account for multiple comparisons, we applied Benjamini-Hochberg false discovery rate (FDR) correction to all primary analyses (88 comparisons) with a threshold of adjusted *P*-values (q) < 0 .10 (q < 0.1 was considered statistically significant).

## RESULTS

### Patient demographics and clinical characteristics

During the study period, 43 consecutive patients underwent living donor ABOi kidney transplantation at West China Hospital, Sichuan University, China. We excluded 15 recipients and their corresponding living donors (pretransplant panel reactive antibody positivity n = 9, preoperative detection of bleeding tendency n = 4, and blood group A2 or A2B subtype n = 2), leaving 28 donor–recipient pairs for inclusion in the study. Of the included donors, 14 (50.0%) were blood group A, 11 (39.3%) were blood group B, and 3 (10.7%) were blood group AB.

Donors were predominantly female (n = 20, 71.4%), whereas recipients were predominantly male (n = 19, 67.9%). The median age was 51.5 years (IQR = 23, 61) for donors and 29.5 years (IQR = 18, 65) for recipients. The median follow-up duration was 307 days (IQR = 123, 459). None of the 28 recipients had undergone previous transplantation (**Table 1**).

**Table 1:** Clinical characteristics of the study population*

	All (n = 28)
**Donor**	
Age in years, Mdn (IQR)	51.5 (23–61)
Male/female, n	8/20
BMI in kg/m^2^, x̄ (SD)	24.68 (3.10)
Blood group	
*A*	14 (50.0)
*B*	11 (39.3)
*AB*	3 (10.7)
*O*	0
Secretor status	
*Secretor*	23 (82.1)
*Weak- or non-secretor*	5 (17.9)
**Recipient**	
Age in years, Mdn (IQR)	29.5 (18–65)
Male/female, n	19/9
BMI in kg/m^2^, x̄ (SD)	20.06 (1.97)
Blood group	
*A*	1 (3.6)
*B*	4 (14.3)
*AB*	0 (0.0)
*O*	23 (82.1)
Pre-transplant PRA†	
*Positive*	4 (14.3)
*Negative*	24 (85.7)
Length of time on dialysis in months, x̄ (SD)	27 (29.1)
Initial IgM titre, Mdn (IQR)‡	32 (4–256)
Initial IgG titre, Mdn (IQR)‡	32 (0–256)
Pretransplant IgM titre, Mdn (IQR)‡	1 (0–8)
Pretransplant IgG titre, Mdn (IQR)‡	4 (0–64)
HLA MM, x̄ (SD)	3.81 (0.96)
DSA presence	0
Pretransplant ATG therapy	4 (14.3)
Repeated kidney transplantation	0 (0.0)
Blood type mismatch (donor/recipient)	
*A/O*	11 (39.3)
*B/O*	11 (39.3)
*A/B*	3 (10.7)
*B/A*	0 (0.0)
*AB/A*	1 (3.6)
*AB/B*	1 (3.6)
*AB/O*	1 (3.6)

ATG – anti-thymocyte globulin, BMI – body mass index, DSA – donor-specific anti-HLA, HLA MM – human leukocyte antigen mismatch, IgG – immunoglobulin G, IgM – immunoglobulin M, IQR – interquartile range, Mdn – median, PRA – panel reactive antibody, SD – standard deviation, x̄ – mean

*Presented as n (%) unless specified otherwise.

†PRA>0 was regarded as positive.

‡IgG/IgM titres were transformed to log base 2.

### Graft ABO blood group antigen expression levels

**Figure 1:**
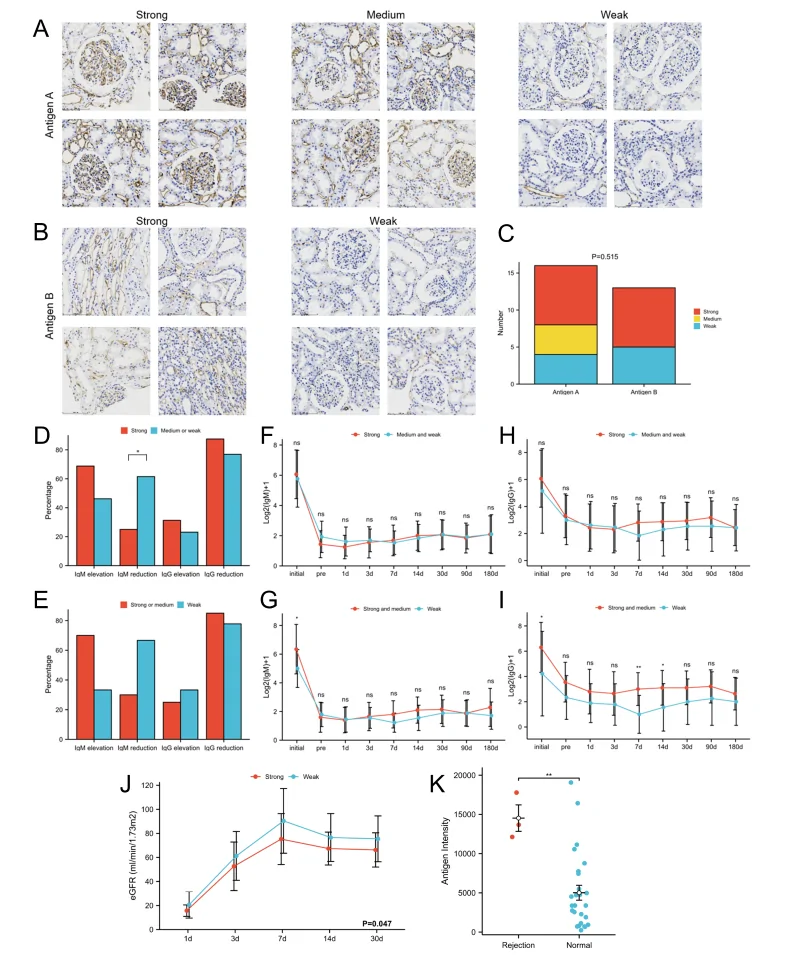
Donor blood group antigen expression, posttransplant antibody dynamics, and their impact on renal function and antibody-mediated rejection. **Panel A.** Renal A antigen expression exhibited three patterns (strong, medium, and weak) across individuals. **Panel B.** Renal B antigen exhibited two patterns (strong and weak) across individuals. **Panel C.** Distribution of blood group antigen expression levels: three patterns (strong, medium, and weak) for antigen A and two patterns (strong and weak) for antigen B. **Panel D.** Comparison of the percentage of patients with titre change between the strong-antigen donor group and the medium/weak-antigen donor group. **Panel E.** Comparison between the strong/medium-antigen donor group and the weak-antigen donor group. **Panel F.** Perioperative changes in IgM. **Panel G.** Perioperative changes in IgG titres in strong *vs.* medium/weak antigen donor groups. **Panel H.** IgM. **Panel I**. IgG in strong/medium *vs.* weak antigen donor groups, shown as line charts. **Panel J.** Generalised estimating equation analysis of renal function after kidney transplantation according to donor blood group antigen expression intensity. Lower eGFR levels were observed in recipients of kidneys from donors with strong antigen expression within the first month post-transplant. **Panel K.** Three recipients developed antibody-mediated rejection (AMR), and their donor antigen expression levels were numerically higher than those in the non-rejection group. *P < 0.05, **P < 0.01.

To assess donor–recipient ABO incompatibility, we detected and semiquantitatively evaluated donor blood group antigen types and the corresponding recipient antibodies by IHC staining. We performed IHC staining for antigen A in 16 donors and for antigen B in 13 donors. Consistent with the findings of a previous study [15], we identified three distinct levels of antigen A expression (designated as strong, medium, and weak) and two distinct levels of antigen B expression (strong and weak) based on the final IHC staining score and staining intensity quantified by image analysis software (**Figure 1**, Panels A–C; Material S3 in the **Online Supplementary Document**).

### Graft ABO blood group antigen expression and outcomes of ABOi kidney transplantation

Based on IHC scoring of graft A/B antigen expression, we classified recipients into strong, medium, or weak blood group antigen level groups to assess correlations with posttransplant fluctuations in anti-A/B antibody titres. We observed an association between graft antigen level and IgM titre elevation or reduction (**Figure 1**, Panels D and E). Compared with recipients of grafts with strong antigen expression, a higher proportion of recipients of grafts with medium or weak expression showed a reduction (70.6% *vs.* 33.3%, rate difference = –0.373; 95% confidence interval (CI) = –0.716, –0.029, χ^2^ = 3.95, uncorrected *P* = 0.047). When the strong and medium expression groups were combined, recipients receiving grafts with strong/medium expression tended to exhibit IgM titre elevation (82.4% *vs.* 50.0%, *P* = 0.064), whereas those receiving grafts with weak expression were observed to show IgM titre reduction (82.4% *vs.* 50.0%, uncorrected *P* = 0.064). In contrast, we found no significant correlation between graft antigen level and IgG titre elevation or reduction. Nevertheless, IgG titres were higher on day seven (difference = 2.00; 95% CI = 1.00, 4.00, *P* = 0.003, remained significant after multiple comparison correction) and on day 14 (difference = 2.00; 95% CI = 0, 3.00, uncorrected *P* = 0.040, exploratory after correction) in recipients of grafts with strong or medium antigen expression (**Figure 1**, Panels H and I). This finding may reflect that AMR mediated by blood group antibodies typically occurs within the first month after ABOi transplantation. We did not observe significant differences in IgM titres according to graft antigen expression level (**Figure 1**, Panels F and G).

We next examined the impact of graft antigen expression level on graft dysfunction, rejection and posttransplant renal function. Compared with recipients of grafts with medium or weak A/B antigen expression, those receiving grafts with strong A/B antigen expression had worse estimated eGFR on postoperative day one (x̄ = 15.25 (SD = 4.45) *vs.* x̄ = 20.99 (SD = 9.91) ml/min/1.73m^2^, uncorrected *P* = 0.047, exploratory after correction). We observed no other significant correlations between graft antigen level and Scr or eGFR (Material S4 in the **Online Supplementary Document**). We then focused on Scr and eGFR values during the period of highest incidence of blood group antibody-mediated AMR, *i.e.* within the first month after surgery [22,23]. Further, GEE analysis showed that posttransplant eGFR during this period was associated with donor graft antigen expression level (weak *vs.* strong regression coefficient = 15.13; 95% CI = 0.172, 30.077, uncorrected *P* = 0.047, exploratory after correction) (**Figure 1**, Panel J), indicating that recipients of weak A/B antigen grafts had better eGFR in the first postoperative month.

Four recipients developed oliguria and delayed reduction in Scr after transplantation, which was defined as graft dysfunction. Furthermore, three recipients were diagnosed with graft rejection; posttransplant biopsy confirmed antibody-mediated rejection, and the donor kidney of this recipient exhibited stronger antigen expression than those of the other recipients (difference = 10,301.8; 95% CI = 4389.5, 14.413.2, uncorrected *P* = 0.0088, exploratory after correction) (**Figure 1**, Panel K). All three recipients who experienced graft rejection had elevated IgM levels and were clinically diagnosed with blood group antigen-mediated rejection.

### Identification of genes associated with graft blood group antigen expression intensity

**Figure 2:**
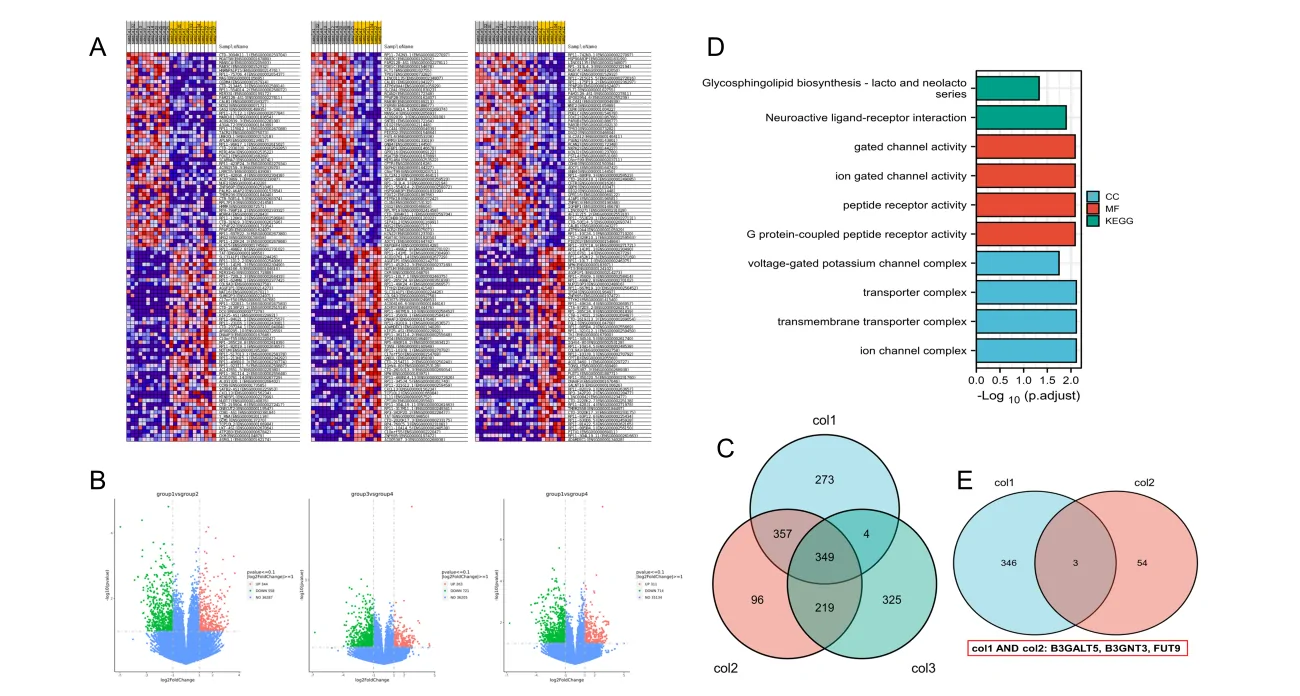
Differentially expressed genes (DEGs) among three subgroup comparisons. **Panel A.** Heatmaps of DEGs in three subgroup comparisons: strong *vs.* medium/weak, strong/medium *vs.* weak, and strong *vs.* weak. **Panel B.** Volcano plots of DEGs in the same three subgroup comparisons. **Panel C.** Venn diagram showing the common DEGs identified across the three subgroups (col1, col2, col3 represent strong *vs.* medium/weak, strong/medium *vs.* weak, and strong *vs.* weak, respectively). **Panel D.** GO/KEGG enrichment analysis of the common DEGs. **Panel E.** Key DEGs involved in the glycolipid synthesis pathway: B3GALT5, B3GNT3, and FUT9.

To screen genes that may influence blood group antigen expression intensity in donor kidneys, we divided sequencing samples into three groups based on IHC staining results – strong, medium, and weak antigen expression. Pairwise comparisons of differentially expressed genes (DEGs) were performed as follows: strong *vs.* medium plus weak; strong plus medium *vs.* weak; and strong *vs.* weak. Using appropriate filtering thresholds, we identified 983, 1021, and 897 DEGs in the three comparisons, respectively. The distribution of DEGs is shown in heatmaps and volcano plots (**Figure 2**, Panels A and B). Venn analysis of the three DEG sets identified 349 common DEGs consistently associated with antigen expression intensity across all comparisons (**Figure 2**, Panel C).

To explore molecular pathways and mechanisms related to graft antigen expression, we performed Gene Ontology (GOO) and Kyoto Encyclopedia of Genes and Genomes (KEGG) enrichment analyses on the 349 common DEGs (**Figure 2**, Panel D). Two KEGG pathways were enriched – glycosphingolipid biosynthesis and neuroactive ligand-receptor interaction. Given that A/B blood group antigens are carbohydrates, we focused on the glycosphingolipid biosynthesis pathway (Table S9 in the **Online Supplementary Document**). Within the glycosphingolipid biosynthesis pathway, we identified three key DEGs – B3GALT5, B3GNT3, and FUT9 (**Figure 2**, Panel E). Of the 349 common DEGs, 90 genes (25.8%) were up-regulated (higher expression in stronger antigen groups), and 259 genes (74.2%) were down-regulated (lower expression in stronger antigen groups). The down-regulated set included B3GALT5, B3GNT3, and FUT9. The absolute log_2_FC of the 349 common DEGs ranged from 1.00 to 4.51, with a median log_2_FC of 2.06 (Material S5 in the **Online Supplementary Document**). The three key genes of interest (B3GALT5, B3GNT3, FUT9) had log_2_FC values of –1.39, –1.45, and –4.01, respectively, indicating moderate to large effect sizes. We selected B3GALT5, B3GNT3, and FUT9 from the glycosphingolipid biosynthesis pathway for further validation because this pathway encodes key enzymes for ABO antigen synthesis, and these three genes showed the largest and most consistent down-regulation among all differentially expressed genes in the pathway [24].

**Figure 3:**
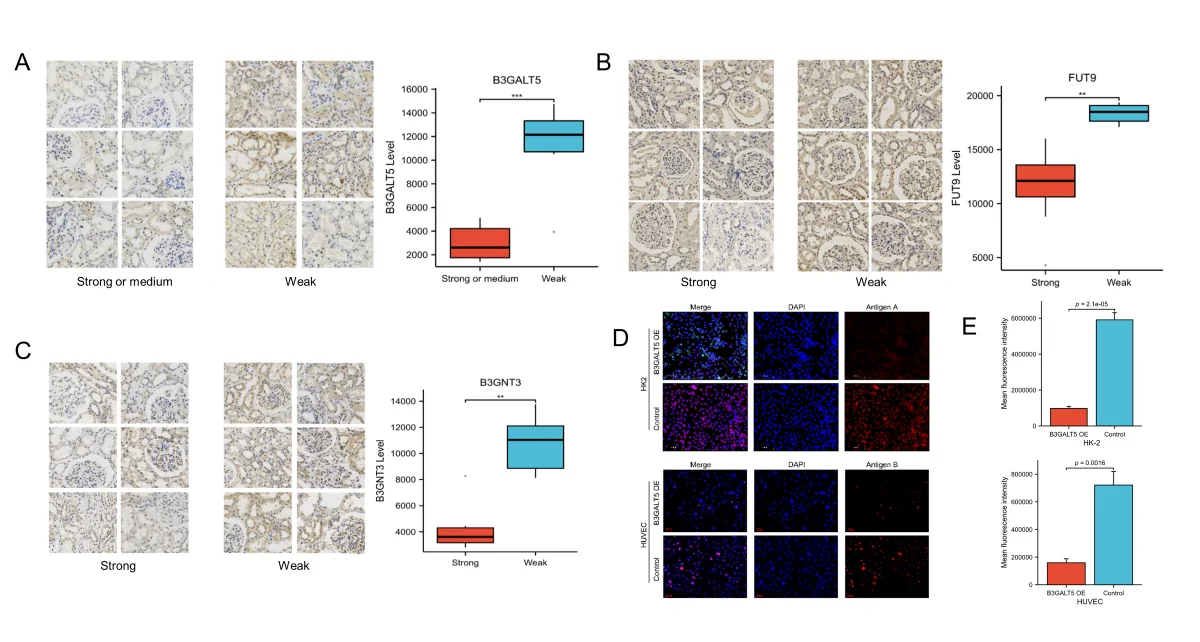
Expression of B3GALT5, B3GNT3, and FUT9 across blood group antigen intensity groups and the effect of B3GALT5 overexpression on antigen A expression. **Panel A.** Expression levels of B3GALT5 protein in groups with different blood group antigen intensities (magnification: 20×). B3GALT5 expression was lower in groups with higher antigen expression. **Panel B.** Expression levels of B3GNT3 protein in groups with different blood group antigen intensities (magnification: 20×). B3GNT3 expression was lower in groups with higher antigen expression. **Panel C.** Expression levels of FUT9 protein in groups with different blood group antigen intensities (magnification: 20×). FUT9 expression was lower in groups with higher antigen expression. **Panel D.** B3GALT5 overexpression significantly reduced A antigen expression in HK-2 cells and B antigen expression in HUVECs. **Panel E.** Quantification of A/B antigen fluorescence intensity in HK-2 and HUVEC cells. OE, overexpression.

We performed IHC staining of donor biopsy tissues for B3GALT5, B3GNT3, and FUT9 and examined correlations with antigen expression levels. Notably, B3GALT5 expression was significantly lower in the strong and medium antigen expression groups (IHC intensity x̄ = 3,005.8 (SD = 1,540.7) *vs.* x̄ = 11,154.8 (SD = 3,846.1); 95% CI = –12,189.8, –4,108.2; t-statistic (t) = –4.87, degrees of freedom (df) = 6.37, *P* = 0.002). Similarly, lower expression levels of B3GNT3 (x̄ = 4,305.77 (SD = 2,027.3) *vs.* x̄ = 10,694.74 8 (SD = 2,135.0); 95% CI = –8,937.46, –3,840.48, *t* = –5.53, df = 10.85, *P* < 0.001) and FUT9 (x̄ = 11,617.17 (SD = 3,744.6) *vs.* x̄ = 18,347.54 (SD = 936.4); 95% CI = –9,898.85, –3,561.89, *t* = –4.88, df = 8.14, *P* = 0.001) were observed in the strong antigen expression group (**Figure 3**, Panels A–C). We used Spearman’s rank correlation analysis to assess the relationship between the expression levels of B3GALT5, B3GNT3, and FUT9 and the IHC intensity of blood group A/B antigens (ranked as weak, medium, and strong). The results revealed significant negative correlations for all three genes: B3GALT5 (Spearman’s rank correlation coefficient (ρ) = −0.784, *P* = 0.002), B3GNT3 (ρ = −0.825, *P* < 0.001), and FUT9 (ρ = −0.796, *P* < 0.001). These findings indicate that higher antigen expression is associated with lower transcript levels of these three glycosyltransferase genes (Material S6 in the **Online Supplementary Document**).

As described in the Methods, we determined the ABO genotype of HK-2 and HUVEC cells by PCR amplification and Sanger sequencing, revealing blood group A (genotype A101/A101) in HK-2 and blood group B (genotype B101/B101) in HUVEC cells. A B3GALT5-overexpressing HK-2 and HUVEC cell model was established, and cells were divided into control and B3GALT5-overexpression groups. qPCR and Western blotting (**Table 2**; Material S7 in the **Online Supplementary Document**) confirmed that B3GALT5 expression was significantly increased at both the mRNA and protein levels in the overexpression group in HK-2 cells. Immunofluorescence staining showed reduced blood group A antigen expression in B3GALT5-overexpressing HK-2 cells compared with controls (**Figure 3**, Panel D). B3GALT5 overexpression led to a significant reduction in A/B antigen fluorescence intensity compared to the control group in HK-2 (mean fluorescence intensity (MFI) x̄ = 977,032.5 (SD = 230,525.1) *vs.* x̄ = 5,910.708.8 (SD = 796,373.1), x̄ reduction = 83.5%; 95% CI = –6,152.563, –3,714.789, *P* < 0.0001) (**Figure 3**, Panel E), and HUVEC cells (MFI x̄ = 158,928.8 *vs.* x̄ = 721,076.2, x̄ reduction = 78.0%; 95% CI = –863,842, –260,453, *P* = 0.0016), suggesting that B3GALT5 overexpression may down-regulate blood group A/B antigen expression in donor kidney tissues.

**Table 2:** Expression changes of B3GALT5 mRNA in human HK-2 cells*

Group	Samples, n	Gene expression level RQ value, x̄ (SD)
Empty vector group (NC)	3	1003 (0.112)
Transfection group	3	478,067 (27,579)

NC –normal control, RQ – relative quantification, SD – standard deviation, x̄ – mean

*The mRNA expression was determined by qRT-PCR and analysed using the 2 – ΔΔCt relative quantification method.

After Benjamini-Hochberg correction for multiple comparisons (FDR = 0.10), only one comparison remained statistically significant – IgG titre at day seven in the strong/medium *vs.* weak antigen group (*P* = 0.0011, q = 0.097). Although the difference in IgG levels at day seven remained statistically significant after multiple-comparison correction, this result should be interpreted with caution because of its borderline significance and the limited sample size; therefore, rigorous validation in a larger, independent cohort is warranted (Table S10 in the **Online Supplementary Document**).

## DISCUSSION

Immunosuppressive strategies have successfully overcome the ABO blood group barrier. Pretransplant recipient anti-A/B titres, which reflect the ability to bind graft blood group antigens and have been confirmed to play a critical role in the incidence of AMR, must be reduced to a safe level to prevent AMR [11,25]. However, the relationship between AMR and anti-A/B titres is not always consistent. Even when pretransplant anti-A/B titres are maintained within a relatively safe range, some grafts still lose function due to AMR, a phenomenon we hypothesise may be influenced by donor A/B antigen levels. To the best of our knowledge, no studies have investigated how A/B antigen expression levels affect the prognosis of ABOi transplantation. In a previous study, we demonstrated that blood group antigen expression varies across tissues and among individuals, and that the expression patterns observed by Western blotting are consistent with those obtained by IHC in kidney and liver tissues [26]. In this study, we examined how A/B antigen expression levels affect post-transplant fluctuations in anti-A/B titres, renal function, graft dysfunction, and graft rejection, and we preliminarily investigated the molecular mechanisms underlying kidney blood group antigen expression.

We identified three distinct expression levels (strong, medium, and weak) in blood group A1 subtype donors. We did not include any patients with the A2 subtype because the A2 subtype is less than 1% in the Chinese population, and its significantly weaker antigenicity may affect IHC staining assessment [27]. Breimer *et al.* also identified three A antigen expression patterns, described as having major, minor, and minimal staining distribution [15]. In kidney tissue with blood group B antigen, we identified two expression patterns (strong and weak) by IHC staining. Similarly, Bentall *et al.* observed different expression levels of blood group B antigen subtypes in ABOi donor kidney tissue [28].

We identified a strong relationship between A/B antigen expression levels and ABOi kidney transplantation prognosis, as reflected by anti-A/B IgM titres, IgG titres, and renal function. The GEE analysis revealed that recipients of weak antigen grafts had a significantly higher eGFR during the first postoperative month compared to recipients of strong antigen grafts (regression coefficient = 15.13 ml/min/1.73m^2^; 95% CI = 0.172, 30.077, uncorrected *P* = 0.047). To our knowledge, a universally accepted minimal clinically important difference for eGFR specifically at one month after kidney transplantation has not been formally established. However, based on expert opinion [29], a difference of >5–10 ml/min/1.73m^2^ is generally considered clinically meaningful in early graft function. Our observed difference of 15.13 ml/min/1.73m^2^ therefore exceeds this threshold, suggesting that the better early renal function in recipients of weak antigen grafts is likely clinically relevant. Nevertheless, given the limited sample size and the fact that this finding did not remain statistically significant after correction for multiple comparisons (Bonferroni-adjusted *P* > 0.05), it should be interpreted as an exploratory observation that warrants validation in larger prospective cohorts.

We also observed a significant correlation between A/B antigen levels and graft rejection. Specifically, three recipients developed AMR within one month post-surgery. Because blood group antibody-mediated rejection most often occurs within the first month after transplantation, and because these cases were accompanied by a rebound in blood group antibody titres, we diagnosed them as blood group antibody-mediated rejection. Notably, blood group antigen levels in the transplanted kidneys of these three recipients were higher than average, suggesting that donor kidney A/B antigen levels may increase the risk of blood group antibody-mediated rejection. Our findings imply that grafts with higher A/B antigen expression are more likely to bind anti-A/B antibodies, thereby increasing the risk of renal tissue damage and leading to post-transplant antibody fluctuations and renal dysfunction.

B3GALT5 encodes a protein that synthesises type I Lewis antigens, which are elevated in certain tumours, and its expression correlates with tumour prognosis [30]. B3GNT3 (also known as B3GALT8) encodes a type II transmembrane N-acetylglucosaminyltransferase involved in the synthesis of poly-N-acetylglucosamine chains and the Lewis antigen backbone [31]. Like B3GALT5, B3GNT3 expression is associated with the prognosis of several tumours [32]; however, no study has linked B3GNT3 expression to blood group antigen levels in kidney tissue. FUT9, a member of the FUT family, encodes a Golgi-localised glycosyltransferase essential for the final step of Lewis antigen X synthesis [33]. Our study is the first to show that B3GALT5, B3GNT3, and FUT9 expression in donor kidney tissue is negatively correlated with A/B blood group antigen levels, and that B3GALT5 overexpression reduces A antigen expression on HK-2 cells. Together with our previous findings, these observations have theoretical importance and strong potential for clinical translation by reducing donor kidney blood group antigens and improving outcomes in ABO-incompatible kidney transplantation.

To our knowledge, this is the first study to explore the relationship between graft ABO blood group antigen expression levels and clinical outcomes in ABOi living donor kidney transplantation. Our results indicate that recipients with higher graft A/B antigen levels are more likely to experience postoperative IgM elevation and poorer graft function. Furthermore, additional analyses showed that changes in IgM anti-A/B antibody levels, driven by differential graft A/B antigen expression, may affect post-transplant prognosis. These findings suggest that IgM anti-A/B antibodies may be associated with rejection. In line with our observations, a previous study reported a high incidence of early acute rejection or thrombotic microangiopathy in A2-to-O kidney transplants with high recipient anti-A IgM titres [34].

In ABOi transplantation, the recipient’s ABO blood group antibodies function as a type of DSA, and ABOi-mediated rejection is AMR driven by blood group antibodies [35]. Post-transplant levels of IgM DSA have been associated with graft failure in ABO-compatible renal transplantation [36] as well as in lung transplantation [37]. Moreover, IgM antibody levels are correlated with disease severity and prognosis in autoimmune diseases such as primary biliary cirrhosis and multiple sclerosis [38,39]. In ABOi renal transplantation, anti-A/B antibodies target endothelial cells. A possible explanation for immune adaptation is that endothelial cells resist complement-mediated damage through various properties of the cell membrane and cellular metabolism [25]. The surface of endothelial cells is decorated with acidic sugars, such as heparan sulphate, and complement regulatory proteins that slow and may block complement activation [40]. In addition, endothelial cells can actively process complement activation products, altering injury kinetics and allowing potential repair [41–43]. In most ABOi graft recipients, the lower density of bound IgM on the graft may be insufficient to overcome these endothelial defences, which could explain the establishment of immune tolerance and the absence of hyperacute rejection in most cases.

However, this study has several limitations. First, it was a prospective analysis conducted at a single institution, and the follow-up duration was relatively short. Nevertheless, because blood group antibody-mediated rejection most likely occurs within the first month after ABOi kidney transplantation, we focused primarily on changes in antibody titres and renal function during the first six months post-transplant. The relationship between donor kidney antigen expression levels and long-term outcomes in ABOi kidney transplantation still requires further investigation. Second, the sample size was limited. Third, the IHC staining was rated semiquantitatively and may have been subject to observer bias. Furthermore, while the regulatory effect of B3GALT5 on A/B antigen expression was confirmed in both HK-2 and HUVEC cells, the primary translational relevance for AMR remains the endothelium; future studies using primary renal microvascular endothelial cells are warranted.

## CONCLUSIONS

We identified three distinct expression patterns for antigen A (strong, medium, and weak) and two for antigen B (strong and weak). Recipients of grafts with strong A/B antigen expression were more likely to show an increase in IgM titres (exploratory finding, uncorrected *P* = 0.047). Furthermore, recipients with weak A/B antigen grafts may have better eGFR during the first month post-transplant than those with strong antigen grafts. Notably, kidney antigen levels may be associated with blood group antibody-mediated rejection and with the expression of B3GALT5, B3GNT3, and FUT9. Notably, B3GALT5 overexpression reduces blood group antigen expression.

## Additional material


Online Supplementary Document

## Data Availability

**Data availability:** The data sets used and/or analysed during the current study are available from the corresponding author on reasonable request.
