# Supplementary material for: B3GALT5 regulates renal A/B antigen levels: a determinant of prognosis and rejection in ABO-incompatible kidney transplantation
Source: J Glob Health. 2026 Sep 25;16:04254. doi: 10.7189/jogh.16.04254 (PMC13613293; doi:10.7189/jogh.16.04254)
Supplement: Supplementary file 1 [file jogh-16-04254-s001.pdf]

Supplement to: Fan Z, Saifu Y, Yang X, Tao L, Xianding W. B3GALT5 regulates renal A/B antigen levels, a determinant of prognosis and rejection in ABOi kidney transplantation. J Glob Health. 2026;16:04254.

Supplementary material 1: Antigen intensity evaluation

The distribution of positive cells was graded as follows: none (not stained) = 0, focal (less than one-third of cells of the same type) = 1, multifocal (less than two-thirds of cells of the same type) = 2, and diffuse (more than two-thirds of cells of the same kind) = 3. The intensity of staining was graded as none (not stained) = 0, slight (light yellow) = 1, mild (brown) = 2, and strong (dark brown) = 3. The final score was the sum of the distribution and intensity scores. Donor grafts were grouped according to A/B antigen expression level based on the final score. Moreover, this assessment of ABO antigen expression was verified by using image analysis software ImageJ (https://imagej.nih.gov/ij/) and Immunohistochemistry Image Analysis Toolbox (https://imagej.nih.gov/ij/plugins/ihc-toolbox/index.html).

Supplementary material 2: Transcriptome sequencing

1. Number of samples per subgroup and selection basis

From the 28 donor-recipient pairs, zero-hour graft biopsies were selected for RNA-seq based on immunohistochemical (IHC) staining intensity of A or B antigens, as described in the main Results. Specifically:

A antigen (16 donors): strong (n=8), medium (n=4), weak (n=4).

B antigen (13 donors): strong (n=8), weak (n=5).

All available samples within each subgroup were sequenced. A total of 23 samples were included in the transcriptome sequencing analysis, including 19 from the original 28 donor-recipient pairs and 4 additional renal tissues from tumor patients to explore the regulatory mechanisms of blood group antigens in the kidney. The remaining samples did not pass quality control and were therefore excluded from further analysis. Number of samples per subgroup subjected to sequencing were shown in Supplementary table 3 (A antigen strong n=6, medium n=3, weak n=4; B antigen strong n=5, weak n=1);.

2. Library preparation protocol

Total RNA was quality-controlled using an Agilent 5400 fragment analyzer and Nanodrop spectrophotometer. mRNA was enriched using Oligo(dT) magnetic beads, fragmented in fragmentation buffer, and reverse-transcribed into first-strand cDNA with random hexamers and M-MuLV reverse transcriptase. Second-strand cDNA was synthesised with RNase H and DNA polymerase I. After end-repair, A-tailing, and adapter ligation, cDNA fragments of 370–420 bp were size-selected using AMPure XP beads. The library was then amplified by PCR and purified again. Library quality was assessed by Qubit 2.0 (initial quantification), Agilent 5400 (insert size), and qRT-PCR (effective concentration >1.5 nM). This detailed protocol is now in the revised Methods.

3. Normalisation procedures

Raw read counts were normalised using the median-of-ratios method implemented in DESeq2 (v1.20.0), which accounts for library size and RNA composition. For descriptive purposes, we also calculated FPKM (Fragments Per Kilobase per Million mapped reads) using featureCounts (v1.5.0-p3). The normalisation steps have been clarified.

4. Sequencing depth

Paired-end sequencing (150 bp × 2) was performed on an Illumina NovaSeq 6000 platform. The average raw read count per sample was 43.6 million (range: 40.9–47.1 million), and the average clean read count after filtering was 42.1 million (range: 39.6–45.7 million). The average clean data output was 6.5 Gb per sample. Detailed per-sample values are provided in Supplementary Table 2.

5. Quality control metrics

Raw reads were filtered to remove adapter-containing reads, reads with ambiguous bases (N), and low-quality reads (where >50% of bases had a Phred score ≤5). For the clean data, Q20 averaged 97.1% (range 96.0–98.0%), Q30 averaged 92.7% (range 90.2–94.4%), and GC content averaged

48.5% (range 46.3–50.3%). The average unique mapping rate to the reference genome was 92.4% (range 88.1–94.5%). All values exceed standard thresholds for high-quality RNA-seq data.

Supplementary material 3: IHC staining intensity quantitated by image analysis software.

ID	Intensity	Intensity group	Blood group A/B
1	12121.7	Strong	A
4	8768.7	Strong	A
9	13675.2	Strong	A
15	16426.4	Strong	A
20	17779.2	Strong	A
25	19065.7	Strong	A
26	11139.3	Strong	A
28	10558.4	Strong	A
5	7732.2	Medium	A
13	4521.0	Medium	A
17	4705.4	Medium	A
27	7446.0	Medium	A
6	926.4	Weak	A
16	3380.8	Weak	A
21	716.2	Weak	A
22	2271.5	Weak	A
3	4970.9	Strong	B
8	3366.0	Strong	B
10	1890.0	Strong	B
12	4745.8	Strong	B
14	5485.3	Strong	B
18	2734.2	Strong	B
23	3383.5	Strong	B
24	2548.8	Strong	B
2	1107.8	Weak	B
7	233.8	Weak	B
11	797.1	Weak	B
17	668.4	Weak	B
19	704.3	Weak	B

Supplementary material 4: Perioperative renal function, graft dysfunction, and rejection. Line chart of perioperative fluctuation of Scr (A) and eGFR (B) level in strong antigen donor group vs. medium or weak antigen donor group. Perioperative fluctuation of Scr (C) and eGFR (D) are compared between the strong or medium antigen donor group and the weak antigen donor group as line charts.

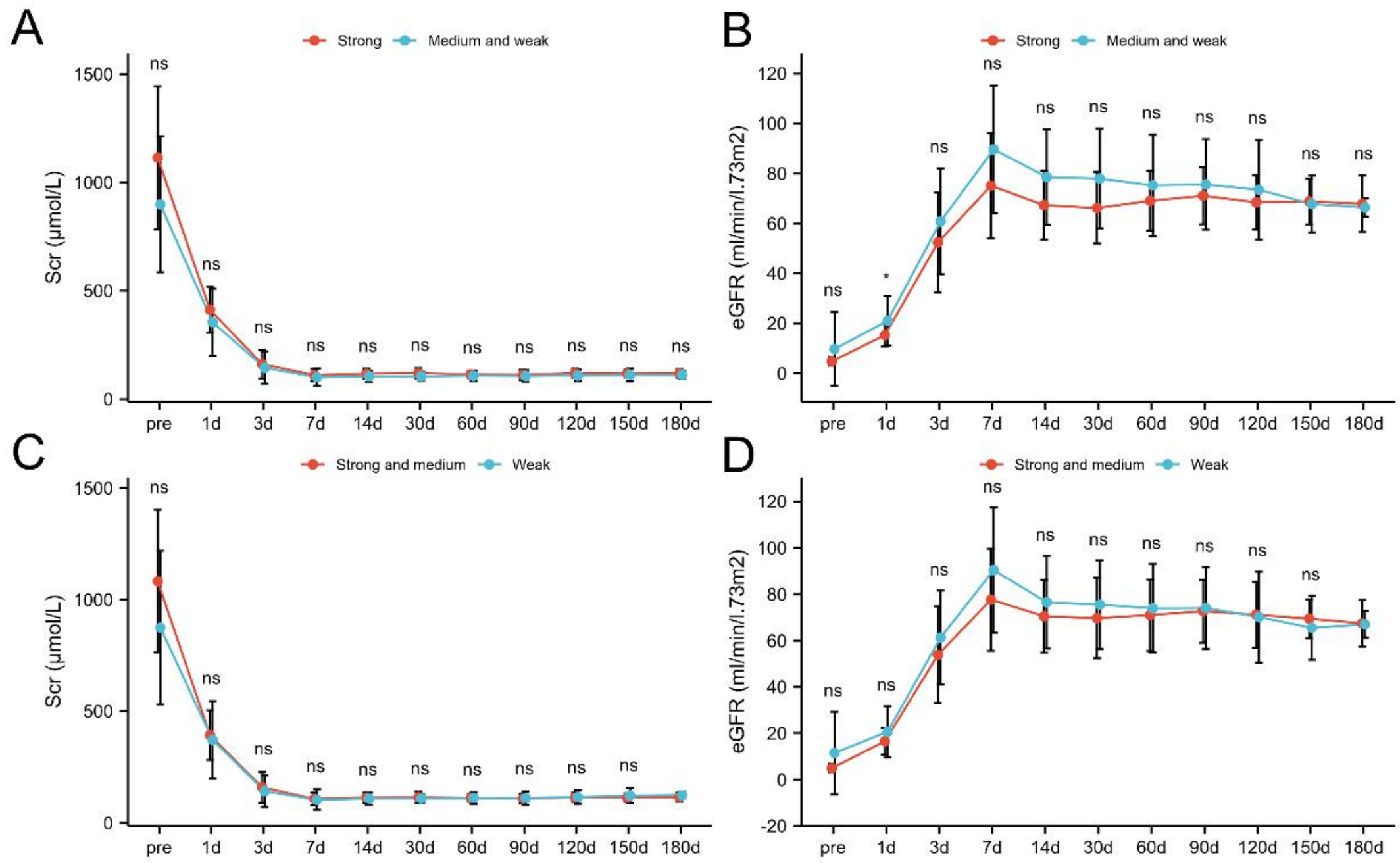

Supplementary material 5: Density plot of log_2FC distribution of the 349 common DEGs

Gene	log2FC	Gene	log2FC	Gene	log2FC	Gene	log2FC	Gene	log2FC
RP11-742N3.1	2.803	NYAP1	-2.31	LINC00323	-1.632	RP11-141M1.3	-2.724	ELFN2	-1.523
COL9A3	-1.623	RP11-10L7.1	-2.833	HIGD1B	-2.18	ZDHHC8P1	-2.14	IGFBP1	2.174
AP000696.2	-1.951	PLIN1	-1.632	SSTR5	-2.349	MLPH	-1.494	RP11-10A14.5	-1.925
AC010761.14	-3.342	NTS	-3.223	IL11	-2.416	RP11-89B16.1	-2.72	HELT	-1.522
CYSLTR2	-1.431	hsa-mir-7162	-1.6	CPT1B	-1.452	GIPR	-1.498	CNTD2	-1.806
RP11-536O18.2	-3.453	AC013460.1	-2.271	ABCA4	-1.533	BCAS1	-2.714	AGGF1P1	-3.847
P2RX2	-3.115	KCNJ5	-1.626	COL17A1	-2.547	IGF2	-1.409	AC011747.6	-2.958
SMOC1	-1.743	GGT2	-1.522	RAB3C	1.985	LRRC55	1.917	CTD-2035E11.4	-2.184
LINC00461	-2.054	ENO1-AS1	-2.185	PLEKHG4B	-1.758	TNNT3	-2.38	VGLL1	-1.624
CNNM1	-1.995	RP11-65L3.2	-3.335	LL09NC01-139C3.1	-1.633	AP001628.6	-2.346	RP11-473M20.5	-2.052
RP11-195B3.1	-2.519	LRCOL1	-1.516	FAM27E1	-2.248	OLFM2	-1.835	SLC44A5	-1.754
RP11-452K12.3	-2.544	SFTA2	-1.692	NUP210P3	-3.978	RDH8	-4.512	SULT2B1	-1.742
MYLK3	1.728	KB-1269D1.8	-2.796	RP11-573D15.9	-1.491	SCXB	-3.463	SULT4A1	2.16
MAB21L2	-3.081	HRK	-3.041	CEACAM7	-2.139	C6orf99	1.588	UNCX	-1.9
ANKRD2	-1.872	CCDC64B	-1.127	SGCG	-1.622	TPSG1	-1.679	AC005387.3	-3.086
NOTUM	-2.099	CLIC5	1.295	RP11-317M11.1	-1.931	CD36	-1.366	AP000344.4	-2.308
RP11-379K22.2	-2.699	C10orf55	-1.929	WNT10A	-1.64	CTD-2020K17.3	-2.153	AP000697.6	-1.767
ATP2B3	-2.203	TFAP2C	-1.668	MAG	1.589	LINC00707	-2.762	FREM3	2.171
FUT9	-4.008	PLET1	-3.464	ADAMTS6	-1.414	ALX1	-1.742	RP11-92G12.3	2.514
WNT11	-1.329	SLC17A9	-1.363	CXCL13	-4.132	RP1-178F15.4	-1.883	RP11-123K19.1	1.975
UTS2R	-3.185	AC004166.6	-1.582	RP11-875O11.1	-1.345	RP11-103J8.1	-2.388	CTD-2231H16.1	-1.873
RP11-74E22.6	-2.321	CTD-3105H18.18	-2.927	AC099552.3	-2.017	RP11-552F3.10	-1.313	IRX5	-1.454
RP11-677M14.2	-1.671	RP11-345J4.5	-2.193	C3P1	-4.433	FAM35BP	-2.053	RP11-566K19.5	-1.402
RP11-49K24.4	-2.502	RP11-554A11.4	1.685	CAPN6	-1.72	MTND4P20	1.779	KCNQ5	1.913
CEP170P1	-1.824	FAP	-1.805	NEFL	-1.491	CACNG6	-3.448	ACOT1	1.548
AC018738.2	-1.532	POU3F1	-1.701	CTC-490E21.10	-1.605	LINC00844	-2.737	RP11-463O9.1	-2.88
IGHV5-78	-2.281	AC107057.1	-2.24	RP11-329B9.4	-1.651	EIF3CL	-1.832	RPL21P135	1.849
RP1-265C24.8	-1.851	RP1-137K24.1	1.645	CTB-114C7.3	-3.034	AC004069.1	3.005	CTRB2	2.848
MANSC4	2.898	COL22A1	1.526	GREB1L	-1.817	VN2R19P	-2.374	SCARNA9	1.929
RP11-267J23.4	-1.348	C1QL4	-1.594	RP11-167N4.2	-2.901	RP11-14I17.2	3.236	RP11-451G4.	1.617

								3	
CMP21-97G8.1	1.656	CTD-2044J15.1	-2.242	CTB-175P5.4	-1.859	IGKV3-15	-1.68	CDC20P1	1.865
AC005775.2	-1.979	CTD-3236F5.1	-2.142	AL592188.2	2.436	MCIDAS	-3.811	RP11-332H14.1	1.456
LGALS12	-2.804	TMEM151B	-2.445	MMP21	-1.585	CDRT15P1	-1.674	SCEL	-3.248
LINC01152	-3.746	ADAMDEC1	-3.008	RP11-107K17.1	-2.055	IGHV4-80	-2.813	GJB3	-2.712
AC069277.2	3.162	RP11-667M19.10	-2.988	CKM	-2.058	FZD10-AS1	-1.432	TMEM72-AS1	-1.55
RP11-65J21.4	-3.131	AC007382.1	1.61	RP11-545A16.3	1.446	CTB-31N19.3	1.252	GALR1	-2.226
PROX1-AS1	-1.69	HIST2H2BF	-2.952	AC074183.4	-2.506	ANGPTL6	-1.433	CTB-23I7.1	3.383
SLCO1A2	-2.506	RP11-561N12.7	-2.254	RP11-157L3.1	-1.621	TIMP4	-1.49	MIR149	-2.618
RP11-524D16__A.3	-1.517	RP1-63G5.5	-1.9	CTB-50E14.5	2.067	CTD-2083E4.5	2.174	TCEAL5	-2.104
RP11-504A18.1	2.189	MFI2	-1.529	CA5A	2.823	RP11-7O14.1	-1.629	C11orf89	-1.993
RP11-499E18.1	-1.408	CTD-2192J16.11	-1.605	KIF25-AS1	-4.027	TULP1	-1.81	NANOGNBP3	-3.65
RP1-170O19.21	-1.901	RP11-686O6.1	-3.316	CTB-174O21.2	-3.358	ZNF890P	-2.311	PLA2G7	2.174
RP11-850F7.7	-3.069	SAA1	-2.789	FGF13-AS1	1.997	OMP	2.096	DMC1	-1.911
PAGE5	-2.545	GPA33	-3.41	RP11-567G11.1	2.776	RP11-757O6.4	3.426	Y_RNA	1.451
RP11-364L4.1	-3.257	IGLV1-36	-2.87	FAM212B-AS1	2.047	CTC-510F12.4	-2.859	DARC	-1.506
LINC00942	2.172	FAM207BP	1.33	RP3-406P24.4	-2.023	CRYGN	-1.852	RP11-82O19.1	-2.801
CTC-1337H24.1	-2.263	LCN1P1	-1.734	AC139887.4	-1.472	RP4-760C5.3	-2.127	TEX35	-2.367
CTD-3064M3.4	-2.722	DNAH11	-1.614	RP11-215N21.1	2.725	SSTR3	1.407	SLC6A3	-2.205
MB	2.212	RP11-361I14.2	-3.22	IL21R	1.449	RP11-62C7.2	2.859	AL590762.7	2.831
SCOCP1	2.509	RP11-586L23.1	-1.743	CTC-260E6.8	-1.837	HCG22	3.212	AC104809.4	-1.727
GGTLC2	-3.719	SCARA5	-1.663	RP11-262H14.5	-1.852	AL445665.1	2.553	MIR331	3.146
FGL1	-2.932	DKFZP667F0711	-1.745	RPL7P19	2.646	AC018755.17	-2.723	RP11-307N16.6	2.165
AP001626.2	-1.275	RN7SL805P	-1.664	B3GNT3	-1.45	SCARNA7	3.41	CTD-2507G9.1	2
RP11-330M19.1	-1.487	WDR64	1.364	RP11-125D12.2	-3.6	RP1-128M12.3	-2.444	RP11-504P24.5	2.359
RPL7AP10	-1.905	OR2A1	-2.969	NEXN-AS1	1.427	C5orf64	1.993	RP11-507K13.6	-2.08
RP11-423F24.3	1.475	RP11-417F21.1	1.374	CCR9	-1.691	TMEM56-RWDD3	-1.679	WNT7B	-3.092
RP11-702H23.2	-1.461	LRRC38	-2.038	RP11-377D9.3	-2.472	DPYSL4	-1.75	HYDIN2	-3.21
EPO	-1.982	AC097381.1	-1.739	LOXHD1	-1.298	RP11-360F5.1	1.568	RP5-1184F4.5	-1.531

RP11-239L20.6	-3.212	AP000295.10	-2.229	C9orf153	2.561	CTD-2529O21.2	-1.782	CTD-2246P4.1	-3.042
CTB-46B19.2	2.199	GSDMA	1.847	B3GALT5	-1.391	ALX4	-1.83	MIR199A1	-2.591
AC073130.3	-3.113	HOXB-AS4	-1.537	KARSP2	-1.943	GABRA5	2.044	IL5	2.804
RP1-86C11.7	-3.34	CTD-2587H24.10	2.377	AC012354.6	-2.394	RP11-52A20.2	2.397	RP11-554D14.2	1.659
RP11-697E22.3	1.706	RP11-696F12.1	-3.078	L1TD1	-2.121	HSD17B2	-1.986	RP11-316K19.3	-2.835
AC090044.2	-1.505	RP11-712C7.1	-2.364	RP11-332K15.1	-2.025	RP3-511B24.6	-2.357	AC092839.3	2.716
CBLN2	-2.543	RNU5B-3P	2.331	AC005082.1	3.103	MEOX1	-2.388	RN7SL838P	-2.056
CTD-2609K8.3	-2.419	RP11-428G5.5	-2.776	PALM2-AKAP2	2.678	RP11-115N12.1	2.428	GLRA3	2.929
RP11-613D13.4	-1.669	RP1-29C18.9	-1.84	AC012499.1	1.228	SPATC1	-1.535	RIMKLBP1	-2.537
SNORD116-1	-2.894	RP11-615I2.2	2.517	RPS4XP6	-3.011	UNC5B-AS1	1.53	KCNS2	-2.783
AC068138.1	-2.62	PF4V1	-1.363	ERVW-1	-1.696	RN7SKP23	1.434	RP11-748H22.1	1.531
VWA3B	-1.999	RP11-367F23.1	1.327	RP11-177C12.4	1.927	snoU13	-2.761		

Supplementary material 6: Spearman's rank correlation between the expression levels of B3GALT5, B3GNT3, FUT9 and the immunohistochemical (IHC) intensity of blood group A/B antigens

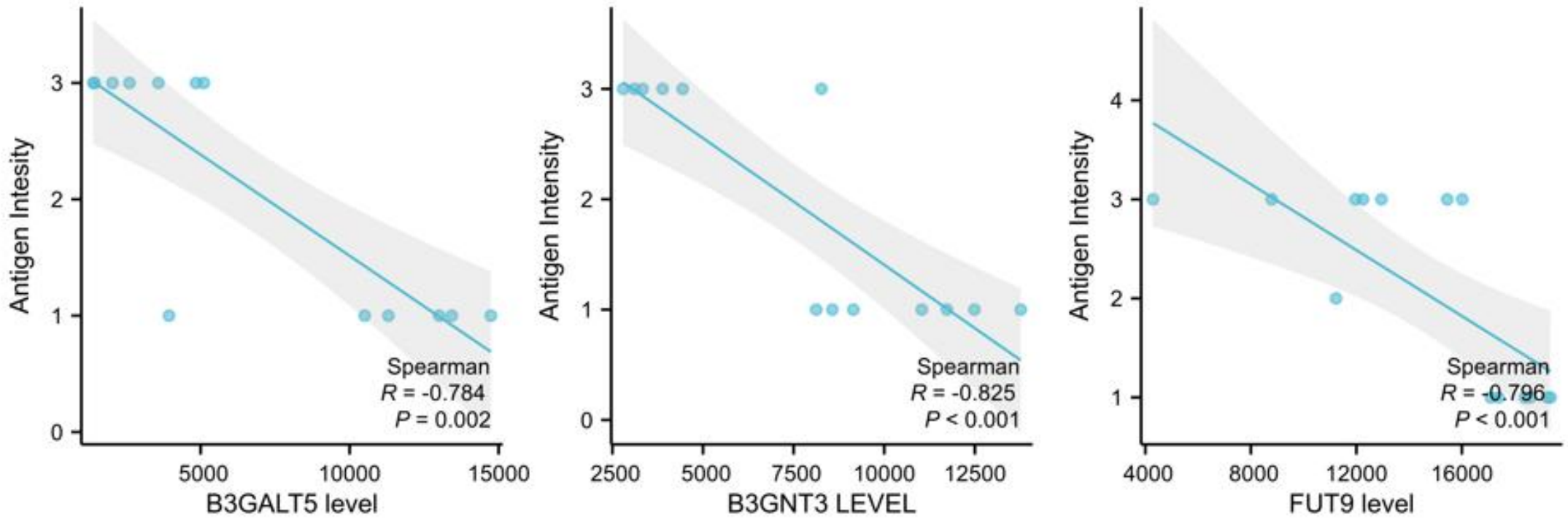

Supplementary material 7: B3GALT5 expression was significantly increased at protein levels in the overexpression group in HK2 cells

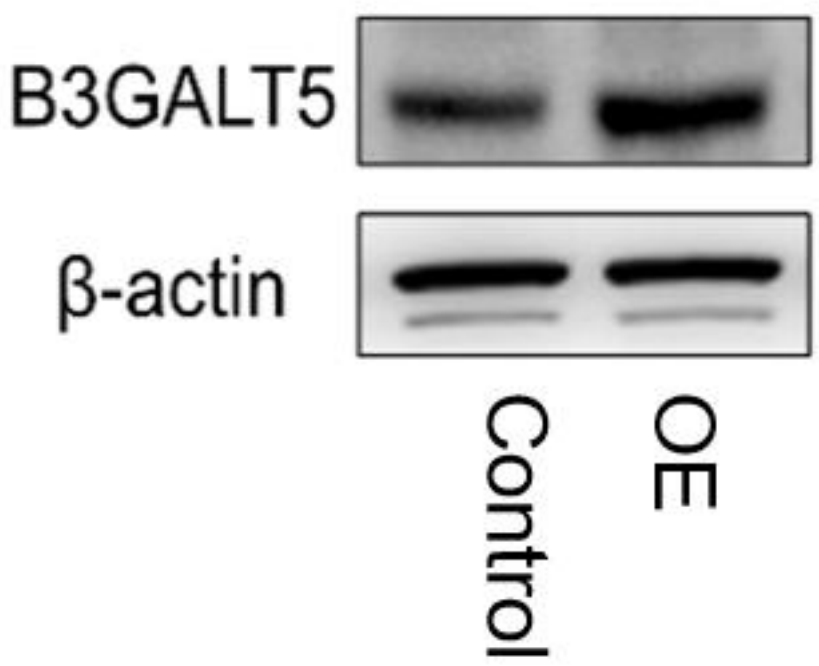

Supplementary Table 1. Quality control results of mRNA transcriptome sequencing samples

Sample	Clean reads	Clean bases	Error rate	Q20	Q30	GC pct
Sample1	47134456	7.07G	0.03	96.97	92.07	49.2
Sample2	44572998	6.69G	0.03	97.22	92.57	49.66
Sample5	40968016	6.15G	0.03	97.38	92.96	49.20
Sample102	41700400	6.26G	0.03	97.36	92.88	49.59
Sample101	43997386	6.60G	0.03	97.21	92.60	49.34
Sample7	45673864	6.85G	0.03	97.14	92.45	49.37
Sample6	42353992	6.35G	0.03	97.36	92.91	49.72
Sample4	41788112	6.27G	0.03	97.17	92.47	47.80
Sample10	44016822	6.60G	0.03	97.96	94.15	49.54
Sample103	45356280	6.80G	0.02	98.00	94.23	50.30
Sample9	41460358	6.22G	0.02	98.00	94.20	49.34
Sample11	43873658	6.58G	0.03	97.96	94.17	49.37
Sample104	43227870	6.48G	0.03	97.88	93.95	49.80
Sample12	43924962	6.59G	0.02	98.02	94.44	49.74
Sample14	42271620	6.34G	0.02	97.97	94.30	49.54
Sample17	45050120	6.76G	0.03	97.01	91.96	48.12
Sample22	43597216	6.54G	0.03	96.2	90.55	47.02
Sample21	42167172	6.33G	0.03	96.06	90.41	47.19
Sample20	40945036	6.14G	0.03	96.00	90.21	47.95
Sample26	43700076	6.56G	0.03	97.20	92.47	46.67
Sample28	43518134	6.53G	0.03	97.00	92.05	48.82
Sample27	44453024	6.67G	0.03	97.23	92.58	49.36
Sample25	45454536	6.82G	0.03	97.27	92.55	46.25

Supplementary Table 2. Quality statistics of reads after filtration

Sample	Total reads	Total mapping	Unique mapping
Sample1	47134456	44970193(95.41%)	42658225(90.50%)
Sample2	44572998	42777700(95.97%)	41091955(92.19%)
Sample5	40968016	39725914(96.97%)	38701822(94.47%)
Sample102	41700400	40328406(96.71%)	39220647(94.05%)
Sample101	43997386	42559862(96.73%)	41453977(94.22%)
Sample7	45673864	44080098(96.51%)	42915853(93.96%)
Sample6	42353992	40940916(96.66%)	39913645(94.24%)
Sample4	41788112	40399877(96.68%)	39231010(93.88%)
Sample10	44016822	42577094(96.73%)	41386901(94.03%)
Sample103	45356280	43897319(96.78%)	42474422(93.65%)
Sample9	41460358	40119950(96.77%)	38947502(93.94%)
Sample11	43873658	42341038(96.51%)	41145693(93.78%)
Sample104	43227870	41733635(96.54%)	40426338(93.52%)
Sample12	43924962	42481034(96.71%)	40842658(92.98%)
Sample14	42271620	39210542(92.76%)	37249230(88.12%)
Sample17	45050120	42996302(95.44%)	41367505(91.83%)
Sample22	43597216	41528728(95.26%)	38821648(89.05%)
Sample21	42167172	39926373(94.69%)	37207217(88.24%)
Sample20	40945036	38876109(94.95%)	36562251(89.30%)
Sample26	43700076	42094595(96.33%)	40895741(93.58%)
Sample28	43518134	41514679(95.40%)	39654196(91.12%)
Sample27	44453024	42578962(95.78%)	41133423(92.53%)
Sample25	45454536	44019920(96.84%)	42820572(94.21%)

Supplementary Table 3. number of samples per subgroup subjected to sequencing

Sample	Blood group antigen type	Intensity	IHC	mRNA sequencing
ABOi-1	A	Strong	√	√
ABOi-2	B	Weak	√	√
ABOi-3	B	Strong	√	NA
ABOi-4	A	Strong	√	√
ABOi-5	A	Medium	√	√
ABOi-6	A	Weak	√	√
ABOi-7	B	Weak	√	√
ABOi-8	B	Strong	√	NA
ABOi-9	A	Strong	√	√
ABOi-10	B	Strong	√	√
ABOi-11	B	Weak	√	√
ABOi-12	B	Strong	√	√
ABOi-13	A	Medium	√	NA
ABOi-14	B	Strong	√	√
ABOi-15	A	Strong	√	NA
ABOi-16	A	Weak	√	NA
ABOi-17	A	Medium	√	√
ABOi-17	B	Weak	√	√
ABOi-18	B	Strong	√	NA
ABOi-19	B	Weak	√	NA
ABOi-20	A	Strong	√	√
ABOi-21	A	Weak	√	√
ABOi-22	A	Weak	√	√
ABOi-23	B	Strong	√	NA
ABOi-24	B	Strong	√	NA
ABOi-25	A	Strong	√	√
ABOi-26	A	Strong	√	√
ABOi-27	A	Medium	√	√
ABOi-28	A	Strong	√	√
ABOi-101	A	Medium	√	√
ABOi-102	A	Strong	√	√
ABOi-103	A	Medium	√	√
ABOi-104	A	Weak	√	√

Supplementary Table 4. Genomic DNA Removal Reaction Mix

Reagent	Volume (μl)
5×gDNA Eraser Buffer	2
gDNA Eraser	1
Total RNA	2
RNase Free dH_2O	5
Total	10

Supplementary Table 5. Reverse Transcription Reaction Mix

Reagent	Volume (μl)
5×PrimeScript Buffer 2	4
PrimeScript RT Enzyme Mix I	1
RT Primer Mix	1
RNA	10
RNase Free dH_2O	4
Total	20

Supplementary Table 6. Primer Sequences Used for Detection

Primer Name	Forward (5'→3')	Reverse (5'→3')	Tm (°C)
GAPDH	tgacttcaacagcgacaccca	caccctgttgctgtagccaaa	60
B3GALT5	cagcaagtggtttgtcagtaaa	ctcttggagacattgtacacct	58

Supplementary Table 7. Real-Time PCR Reaction Mix

Reagent	Volume (μl)
2×Real PCR Easy^{TM} Mix-SYBR	10.0
Forward Primer（10μM）	0.8
Reverse Primer（10μM）	0.8
cDNA template (1:5 dilution)	2.0
ddH_2O	6.4
Total	20.0

Supplementary Table 8. Real-Time PCR Thermal Cycling Conditions

Temperature	Time	Notes
95℃	30s	Initial denaturation
95℃	5s	Denaturation
55℃	30s	Annealing
72℃	30s	Extension and fluorescence acquisition

Supplementary Table 9. GO/KEGG terms

Description	Gene Ratio	Adjust P value	Gene ID	Count
ion channel complex	10/138	0.0077	GABRA5/KCNJ5/KCNS2/GLRA3/CLIC5/KCNQ5/CACNG6/OLFM2/LRRC38/LRRC55	10
transmembrane transporter complex	10/138	0.0077	GABRA5/KCNJ5/KCNS2/GLRA3/CLIC5/KCNQ5/CACNG6/OLFM2/LRRC38/LRRC55	10
transporter complex	10/138	0.0077	GABRA5/KCNJ5/KCNS2/GLRA3/CLIC5/KCNQ5/CACNG6/OLFM2/LRRC38/LRRC55	10
voltage-gated potassium channel complex	5/138	0.0178	KCNJ5/KCNS2/KCNQ5/LRRC38/LRRC55	5
potassium channel complex	5/138	0.0225	KCNJ5/KCNS2/KCNQ5/LRRC38/LRRC55	5
cation channel complex	7/138	0.0301	KCNJ5/KCNS2/KCNQ5/CACNG6/OLFM2/LRRC38/LRRC55	7
collagen trimer	4/138	0.0914	COL9A3/COL17A1/COL22A1/C1QL4	4
G protein-coupled peptide receptor activity	7/118	0.0081	GALR1/GIPR/UTS2R/SSTR3/SSTR5/CCR9/CYSLTR2	7
peptide receptor activity	7/118	0.0081	GALR1/GIPR/UTS2R/SSTR3/SSTR5/CCR9/CYSLTR2	7
ion gated channel activity	10/118	0.0081	GABRA5/KCNJ5/KCNS2/GLRA3/P2RX2/CLIC5/KCNQ5/CACNG6/LRRC38/LRRC55	10
gated channel activity	10/118	0.0081	GABRA5/KCNJ5/KCNS2/GLRA3/P2RX2/CLIC5/KCNQ5/CACNG6/LRRC38/LRRC55	10
channel activity	11/118	0.0112	GABRA5/GJB3/KCNJ5/KCNS2/GLRA3/P2RX2/CLIC5/KCNQ5/CACNG6/LRRC38/LRRC55	11
passive transmembrane transporter activity	11/118	0.0112	GABRA5/GJB3/KCNJ5/KCNS2/GLRA3/P2RX2/CLIC5/KCNQ5/CACNG6/LRRC38/LRRC55	11
voltage-gated potassium channel activity	5/118	0.0112	KCNJ5/KCNS2/KCNQ5/LRRC38/LRRC55	5
neuropeptide receptor activity	4/118	0.0112	GALR1/SSTR3/SSTR5/CYSLTR2	4
voltage-gated ion channel activity	7/118	0.0112	KCNJ5/KCNS2/CLIC5/KCNQ5/CACNG6/LRRC38/LRRC55	7
voltage-gated channel activity	7/118	0.0112	KCNJ5/KCNS2/CLIC5/KCNQ5/CACNG6/LRRC38/LRRC55	7
voltage-gated cation channel activity	6/118	0.0112	KCNJ5/KCNS2/KCNQ5/CACNG6/LRRC38/LRRC55	6
ion channel activity	10/118	0.0128	GABRA5/KCNJ5/KCNS2/GLRA3/P2RX2/CLIC5/KCNQ5/CACNG6/LRRC38/LRRC55	10
neuropeptide binding	3/118	0.0132	GALR1/SSTR3/SSTR5	3
substrate-specific channel activity	10/118	0.0136	GABRA5/KCNJ5/KCNS2/GLRA3/P2RX2/CLIC5/KCNQ5/CACNG6/LRRC38/LRRC55	10
potassium channel activity	5/118	0.0294	KCNJ5/KCNS2/KCNQ5/LRRC38/LRRC55	5
receptor ligand activity	10/118	0.0294	EPO/IGF2/IL5/IL11/NTS/PF4V1/SAA1/WNT7B/CXCL13/WNT10A	10
galanin receptor activity	2/118	0.0366	GALR1/CYSLTR2	2
frizzled binding	3/118	0.0399	WNT7B/WNT11/WNT10A	3
estradiol 17-beta-dehydrogenase	2/118	0.0399	HSD17B2/RDH8	2

Description	Gene Ratio	Adjust P value	Gene ID	Count
activity				
potassium ion transmembrane transporter activity	5/118	0.0650	KCNJ5/KCNS2/KCNQ5/LRRC38/LRRC55	5
omega peptidase activity	2/118	0.0650	GGTLC2/GGT2	2
chloride transmembrane transporter activity	4/118	0.0650	GABRA5/SLC6A3/GLRA3/CLIC5	4
inhibitory extracellular ligand-gated ion channel activity	2/118	0.0650	GABRA5/GLRA3	2
HMG box domain binding	2/118	0.0650	MEOX1/ALX4	2
metalloendopeptidase activity	4/118	0.0650	FAP/ADAMTS6/ADAMDEC1/MMP21	4
cation channel activity	7/118	0.0694	KCNJ5/KCNS2/P2RX2/KCNQ5/CACNG6/LRRC38/ LRRC55	7
ligand-gated anion channel activity	2/118	0.0932	GABRA5/GLRA3	2
Neuroactive ligand-receptor interaction	10/ 57	0.0126	GABRA5/GALR1/GIPR/UTS2R/NTS/SSTR3/SSTR 5/GLRA3/P2RX2/CYSLTR2	10
Glycosphingolipid biosynthesis - lacto and neolacto series	3/ 57	0.0471	B3GALT5/B3GNT3/FUT9	3

Supplementary Table 10. Benjamini-Hochberg correction (FDR = 0.10) to all primary analyses listed in the manuscript (including IgM/IgG titers at multiple time points, eGFR, Scr, rejection, and GEE results).

	P value	BH-adjusted q (FDR=0.1)
Strong vs. median or weak		
IgM elevation	0.2192	0.584533333
IgM reduction	0.04694	0.481485714
IgG elevation	0.6243	0.838011429
IgG reduction	0.4533	0.828503704
Strong or median vs. weak		
IgM elevation	0.06363	0.481485714
IgM reduction	0.06363	0.481485714
IgG elevation	0.6423	0.838011429
IgG reduction	0.6338	0.838011429
IgM, Strong vs. median or weak		
Initial	0.6543	0.838011429
pre	0.1866	0.570012903
1d	0.2664	0.669805714
3d	0.7076	0.864844444
7d	0.6666	0.838011429
14d	0.6636	0.838011429
30d	0.9688	0.9869
90d	0.8465	0.93445
180d	0.9869	0.9869
IgG, Strong vs. median or weak		
Initial	0.3603	0.773326829
pre	0.6295	0.838011429
1d	0.7879	0.931671795
3d	0.8213	0.931671795
7d	0.1149	0.50556
14d	0.3748	0.785295238
30d	0.5006	0.828503704
90d	0.3846	0.787088372
180d	0.9837	0.9869
IgM, Strong or median vs. weak		
Initial	0.0471	0.481485714
pre	0.6575	0.838011429
1d	0.9011	0.955383133
3d	0.7997	0.931671795
7d	0.1122	0.50556
14d	0.1447	0.530566667
30d	0.5084	0.828503704
90d	0.9708	0.9869

180d	0.3244	0.731979487
IgG, Strong or median vs. weak		
Initial	0.045	0.481485714
pre	0.0724	0.481485714
1d	0.1931	0.570012903
3d	0.2123	0.583825
7d	0.0011	0.0968
14d	0.0188	0.481485714
30d	0.0766	0.481485714
90d	0.1998	0.570012903
180d	0.4378	0.828503704
GEE analysis of 1 month eGFR	0.047	0.481485714
Rejection	0.0088	0.3872
Scr, Strong vs. median or weak		
pre	0.0854	0.496423529
1d	0.2473	0.640070588
3d	0.5249	0.835057143
7d	0.4551	0.828503704
14d	0.2008	0.570012903
30d	0.0938	0.496423529
60d	0.4635	0.828503704
90d	0.5921	0.838011429
120d	0.3122	0.722989474
150d	0.5933	0.838011429
180d	0.4954	0.828503704
eGFR, Strong vs. median or weak		
pre	0.1953	0.570012903
1d	0.0474	0.481485714
3d	0.2805	0.685666667
7d	0.1039	0.50556
14d	0.0757	0.481485714
30d	0.0762	0.481485714
60d	0.3586	0.773326829
90d	0.4639	0.828503704
120d	0.5004	0.828503704
150d	0.8495	0.93445
180d	0.8071	0.931671795
Scr, Strong or median vs. weak		
pre	0.1256	0.5112
1d	0.6945	0.860788732
3d	0.5456	0.838011429
7d	0.7714	0.929906849
14d	0.6513	0.838011429
30d	0.4613	0.828503704

60d	0.938	0.982666667
90d	0.8758	0.95148642
120d	0.8956	0.955383133
150d	0.5314	0.835057143
eGFR, Strong or median vs. weak		
pre	0.1406	0.530566667
1d	0.0959	0.496423529
3d	0.2987	0.710421622
7d	0.1278	0.5112
14d	0.1829	0.570012903
30d	0.1808	0.570012903
60d	0.4716	0.828503704
90d	0.6438	0.838011429
120d	0.8258	0.931671795
150d	0.6202	0.838011429
